# Meta-Analysis of Dengue Severity during Infection by Different Dengue Virus Serotypes in Primary and Secondary Infections

**DOI:** 10.1371/journal.pone.0154760

**Published:** 2016-05-23

**Authors:** Kuan-Meng Soo, Bahariah Khalid, Siew-Mooi Ching, Hui-Yee Chee

**Affiliations:** 1 Department of Microbiology and Parasitology, Faculty of Medicine and Health Sciences, Universiti Putra Malaysia, Serdang, Selangor, Malaysia; 2 Department of Medicine, Faculty of Medicine and Health Sciences, Universiti Putra Malaysia, Serdang, Selangor, Malaysia; 3 Department of Family Medicine, Faculty of Medicine and Health Sciences, Universiti Putra Malaysia, Serdang, Selangor, Malaysia; 4 Malaysian Research Institute on Ageing, Universiti Putra Malaysia, Serdang, Malaysia; Institute of Tropical Medicine (NEKKEN), Nagasaki University, JAPAN

## Abstract

**Introduction:**

Dengue virus (DENV) infection is currently a major cause of morbidity and mortality in the world; it has become more common and virulent over the past half-century and has gained much attention. Thus, this review compared the percentage of severe cases of both primary and secondary infections with different serotypes of dengue virus.

**Methods:**

Data related to the number of cases involving dengue fever (DF), dengue hemorrhagic fever (DHF), dengue shock syndrome (DSS) or severe dengue infections caused by different serotypes of dengue virus were obtained by using the SCOPUS, the PUBMED and the OVID search engines with the keywords “(dengue* OR dengue virus*) AND (severe dengue* OR severity of illness index* OR severity* OR DF* OR DHF* OR DSS*) AND (serotypes* OR serogroup*)”, according to the MESH terms suggested by PUBMED and OVID.

**Results:**

Approximately 31 studies encompassing 15,741 cases reporting on the dengue serotypes together with their severity were obtained, and meta-analysis was carried out to analyze the data. This study found that DENV-3 from the Southeast Asia (SEA) region displayed the greatest percentage of severe cases in primary infection (95% confidence interval (CI), 31.22–53.67, 9 studies, n = 598, I^2^ = 71.53%), whereas DENV-2, DENV-3, and DENV-4 from the SEA region, as well as DENV-2 and DENV-3 from non-SEA regions, exhibited the greatest percentage of severe cases in secondary infection (95% CI, 11.64–80.89, 4–14 studies, n = 668–3,149, I^2^ = 14.77–96.20%). Moreover, DENV-2 and DENV-4 from the SEA region had been found to be more highly associated with dengue shock syndrome (DSS) (95% CI, 10.47–40.24, 5–8 studies, n = 642–2,530, I^2^ = 76.93–97.70%), while DENV-3 and DENV-4 from the SEA region were found to be more highly associated with dengue hemorrhagic fever (DHF) (95% CI, 31.86–54.58, 9 studies, n = 674–2,278, I^2^ = 55.74–88.47%), according to the 1997 WHO dengue classification. Finally, DENV-2 and DENV-4 from the SEA region were discovered to be more highly associated with secondary infection compared to other serotypes (95% CI, 72.01–96.32, 9–12 studies, n = 671–2,863, I^2^ = 25.01–96.75%).

**Conclusion:**

This study provides evidence that the presence of certain serotypes, including primary infection with DENV-3 from the SEA region and secondary infection with DENV-2, DENV-3, and DENV-4 also from the SEA region, as well as DENV-2 and DENV-3 from non SEA regions, increased the risk of severe dengue infections. Thus, these serotypes are worthy of special consideration when making clinical predictions upon the severity of the infection.

**Systematic Review Registration:**

PROSPERO CRD42015026093 (http://www.crd.york.ac.uk/PROSPERO)

## Introduction

Dengue virus (DENV) infection has become more common and virulent over the past half-century. It is in fact, endemic in over 100 countries in Africa, America, and Asia, with 390 million new dengue fever infections [1] and approximately 12,000 deaths occurring worldwide every year [2].

The WHO classification on severe dengue infection changes overtime. In the 1997 definition, dengue hemorrhagic fever was characterized by having plasma leakage, high fever, hemorrhagic phenomena, hepatomegaly and circulatory failure, whereas dengue shock syndrome was characterized by having narrow pulse pressure (<20 mmHg (2.7 kPa) [3]. Later in 2009, the classification was redefined, in which severe dengue infection was characterized by having plasma leakage, severe bleeding and severe organ impairment [4].

### 1.1 Severity of different serotypes

Dengue virus consists of four serotypes with ≥30% difference in their overall amino acid sequences [5].

Patients can be infected with more than one serotype of dengue virus in their lifetime. Secondary infection with heterologous serotypes is more severe than primary infection, which may be explained by the antibody-dependent enhancement (ADE) theory. Based on this theory, primary infection leads to the formation of serotype-specific antibodies, which confer long-lasting immunity to the infecting serotype, but short-lasting immunity to other unexposed serotypes. Hence, for secondary infection with different serotypes, the antibodies produced are unable to neutralize the virus, but instead form immune complexes with the virus. These immune complexes have higher affinity towards Fc*γ* receptors on the surfaces of macrophages and other cells, and hence, enhance the entry of the virus into these cells, besides allowing viral replication to occur [6]. This theory was reported in a study that involved animal, in which a higher peak viremia was found during secondary infection compared to that during primary infection [7].

In addition to the increased severity of secondary infection, certain serotypes were found to cause more severe infections than other serotypes, even during primary infection. For example, Anantapreecha et al [8] reported that primary infection with DENV-1 caused severe infections (95% CI, 54.57–67.41) compared to other serotypes (95% CI, 4.58–52.96). This suggests that specific dengue serotypes also play a role in causing severe dengue infections. Moreover, when the immunogenic effects of different serotypes of dengue viruses were compared, it was found that the synthesized NS4A, NS4B, and E peptides of DENV-2 and DENV-3 induced higher total cytokine responses, which included TNF-*α* and IFN-*γ*, compared to other serotypes [9]. In contrast, DENV-4 was reported to be less immunogenic [10].

Furthermore, since many studies have shown that different serotypes caused different effects upon the severity of dengue infections, this meta-analysis gathered several published evidences from various regions, durations, as well as sample sizes of studies, and synthesized the combined data to evaluate the severity of dengue infection for different dengue serotypes.

## Methods

### 2.1 Literature search

A systematic review of dengue hemorrhagic fever, dengue shock syndrome or severe dengue infection was undertaken based on the general principles recommended in the Preferred Reporting Items for Systematic Reviews and Meta-analyses (PRISMA) statements.

#### Data sources

A number of relevant articles had been identified via systematic search of MEDLINE from 1946 until present (via Ovid), Non-Indexed Citations (via Ovid), Embase from 1974 up to present (via Ovid), Scopus and PubMed. On top of that, in order to identify comprehensive studies that were not captured by the database searches, the reference lists of the published systematic reviews were manually screened and more articles were successfully retrieved after title and abstract exclusions.

#### Search strategies

A search of human studies in inception from February 11, 2014 until July 22, 2015 was performed by using subject headings and free text terms. A search was performed with the keywords “(dengue* OR dengue virus*) AND (severe dengue* OR severity of illness index* OR severity* OR DF* OR DHF* OR DSS*) AND (serotypes* OR serogroup*)”, according to the MESH terms suggested by PubMed and Ovid.

### 2.2 Inclusion criteria

Cross-sectional studies from all publication years concerning dengue patients from all age groups and regions were included. Articles published only in English were evaluated.

### 2.3 Exclusion criteria

The relevance of papers was determined by evaluating their types, objectives and methods. Reviews that did not contain original research data were excluded, so did proceedings that failed to employ the peer-review process. In addition, papers with objectives to study the effects of recombinant proteins from different dengue serotypes on respondents, as well as studies of patients colonized but not infected with dengue virus, were also excluded. Moreover, studies complicated with other diseases were excluded to ensure that dengue virus is the causative agent for all included cases. Finally, papers that did not separate primary infection cases from secondary infection cases were also excluded.

### 2.4 Data abstraction

#### Study selection

The titles and the abstracts of the literature search were screened by one reviewer for potentially relevant studies based on the eligibility criteria and they were further double-checked by a second reviewer. After excluding duplicate and apparently irrelevant studies, the full text of the remaining studies were read by two reviewers. The reasons for the exclusion of records were documented during full-text screening. Disagreements between the reviewers were resolved by consensus.

#### Data extraction and quality assessment

The following data were extracted independently and in duplicate by two reviewers into a data extraction form: the citation of the study; the number of participants; the characteristics of participants (study population, and age); the study duration; the serotyping method; the WHO classification method; as well as the number of cases that involved dengue hemorrhagic fever, dengue shock syndrome or severe dengue infection caused by different serotypes of the dengue virus. Disagreements were documented and resolved by discussion with a third reviewer; where doubt remained, the authors were contacted for clarification.

### 2.5 Data analysis

Meta-analysis was carried out by using Comprehensive Meta-Analysis (CMA) V3.3.070 software (USA). A fixed effect model and a random effect model were used to calculate the mean effect size in the selected studies without significant heterogeneity (p > 0.1) and with significant heterogeneity (p < 0.1), respectively. Besides, in order to identify factors that contributed to the heterogeneity, Begg’s funnel plot and Egger linear regression were employed to identify the presence of publication bias, with p < 0.05 considered as significant for the presence of publication bias. Additionally, sensitivity analysis or study omission analysis was carried out to locate a single study that was responsible for the heterogeneity.

### 2.6 Definition and Outcomes

Dengue serotypes were confirmed by using either an RT-PCR or an immunofluorescence assay serotyping method. Primary infection is a single infection, whereas secondary infection is an infection that occurs following a primary infection after some time interval. Both serotypes do not exist at the same time. On the other hand, concurrent infection with dual serotypes is defined as an infection with two serotypes of dengue virus that is present simultaneously in a patient. Meanwhile, severe dengue infection is defined as a severe infection, according to the WHO classification from 2009, or dengue hemorrhagic fever or dengue shock syndrome, according to the WHO classification from 1997. There were 2 outcomes that were studied in this meta-analysis. First, the effect size in percentage of severe dengue cases caused by each dengue serotype, subdivided to (1) primary and secondary infection, (2) SEA or non-SEA regions, and (3) 1997 and 2009 WHO dengue classification. Second, the effect size in percentage of secondary dengue infection caused by each dengue serotype subdivided to SEA and non-SEA regions.

## Results

### 3.1 Results of literature search

**Fig 1:**
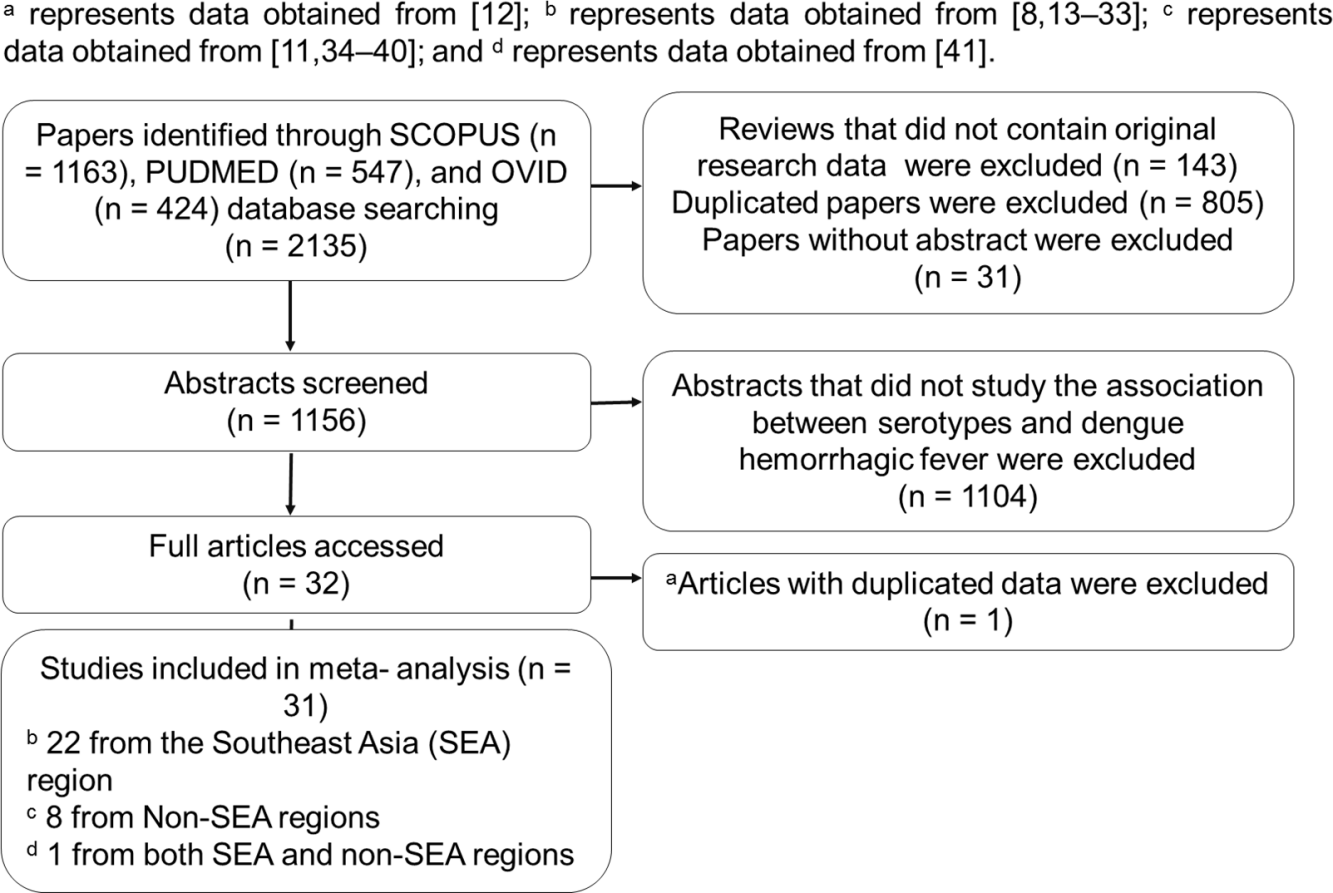
Flow chart of the study selection process. Studies [8,11–41] were cited in this figure.

The study selection process is depicted in Fig 1. The sample size, the study period, the population, the age, and the method of serotyping are presented in Table 1. All the selected studies had been retrospective cross-sectional studies. Thirty-one studies were included for review, with 1 study containing data from both Southeast and non-Southeast Asia regions. Moreover, thirty studies portrayed only the severe DHF caused by different dengue serotypes, which was not specific to the severe DHF caused by primary or secondary infection with dengue serotypes. Out of the 30 authors of the papers contacted, 1 of them, Allonso D [11], responded to provide unpublished data.

**Table 1:** Demographic profile.

Study	Total number of cases included in this meta-analysis	Population	Sample collection year	Age (years)	Serotyping	WHO classification method (year)
[13]	150	Indonesia	1975–1978	0–14	Hemagglutination-inhibition (HI) test	NA
[14]	106	Indonesia	1995–1996	4–9	RT-PCR, PRNT	1997
[15]	8	Indonesia	1975–1978	NA	IF, RT-PCR	NA
[16]	165	Thailand	NA	< 14	RT-PCR	1997
[17]	87	Indonesia	1998	1–78	RT-PCR	1986
[18]	29	Thailand	1998–2000	< 12	RT-PCR, PRNT	1999
[19]	257	Thailand	NA	3–14	ELISA	1997
[20]	5616	Thailand	1973–1999	< 18	NA	1999
[21]	104	Thailand	1998–2000	< 12	RT-PCR	1997
[8]	2715	Thailand	1999–2002	All ages	IF	NA
[22]	99	Thailand	2002	All ages	RT-PCR	1997
[23]	28	Indonesia	2004	1–72	RT-PCR	1999
[24]	20	Philippines	2007–2008	1.5–7 months	RT-PCR	1997
[25]	22	Philippines	2007–2009	3 months	RT-PCR	1997
[26]	167	Vietnam	2006–2008	5–15	RT-PCR	2009
[27]	76	Vietnam	2008	18–31	Real time RT-PCR	1997
[28]	209	Vietnam	2007–2008	17–27	Real time RT-PCR	1997
[41]^a^	99	Cambodia, Vietnam, French, Brazil	2006–2007	All ages	RT-PCR	2009
[29]	394	Thailand	2006–2009	< 14	RT-PCR	1997
[30]	133	Thailand	2006–2008	3–14	RT-PCR	1997
[31]	64	Philippines	2005–2006	7–22	RT-PCR	1997
[32]	134	Thailand	1994–2002	All ages	RT-PCR	1997, 2009
[33]	451	Singapore	2005–2011	18–87	RT-PCR	1997, 2009
[34]	40	Peru	2000–2001	NA	RT-PCR	1997
[42]	3926	Chile, Cuba	1981, 1997	All ages	PRNT	NA
[35]	4	Cuba	2001–2002	NA	RT-PCR, PRNT,	1994 PAHO^c^
[43]	89	Santander, Colombia	1998–2004	NA	IF	1980
[36]	21	France	NA	> 15	RT-PCR	1997
[37]	225	Mexico	2009	NA	RT-PCR	1997
[11]^b^	45	Brazil	NA	All ages	RT-PCR	2009
[38]	258	Brazil	2011	17–64	RT-PCR	2012

^a^ represents paper that contains respondents from both Southeast Asia and non-Southeast Asia (Latin America (Brazil, France)) regions

^b^ represents unpublished data

^c^ represents Pan American Health Organization

NA represents not available

### 3.2 The relationship between dengue serotypes and disease severity

**Fig 2:**
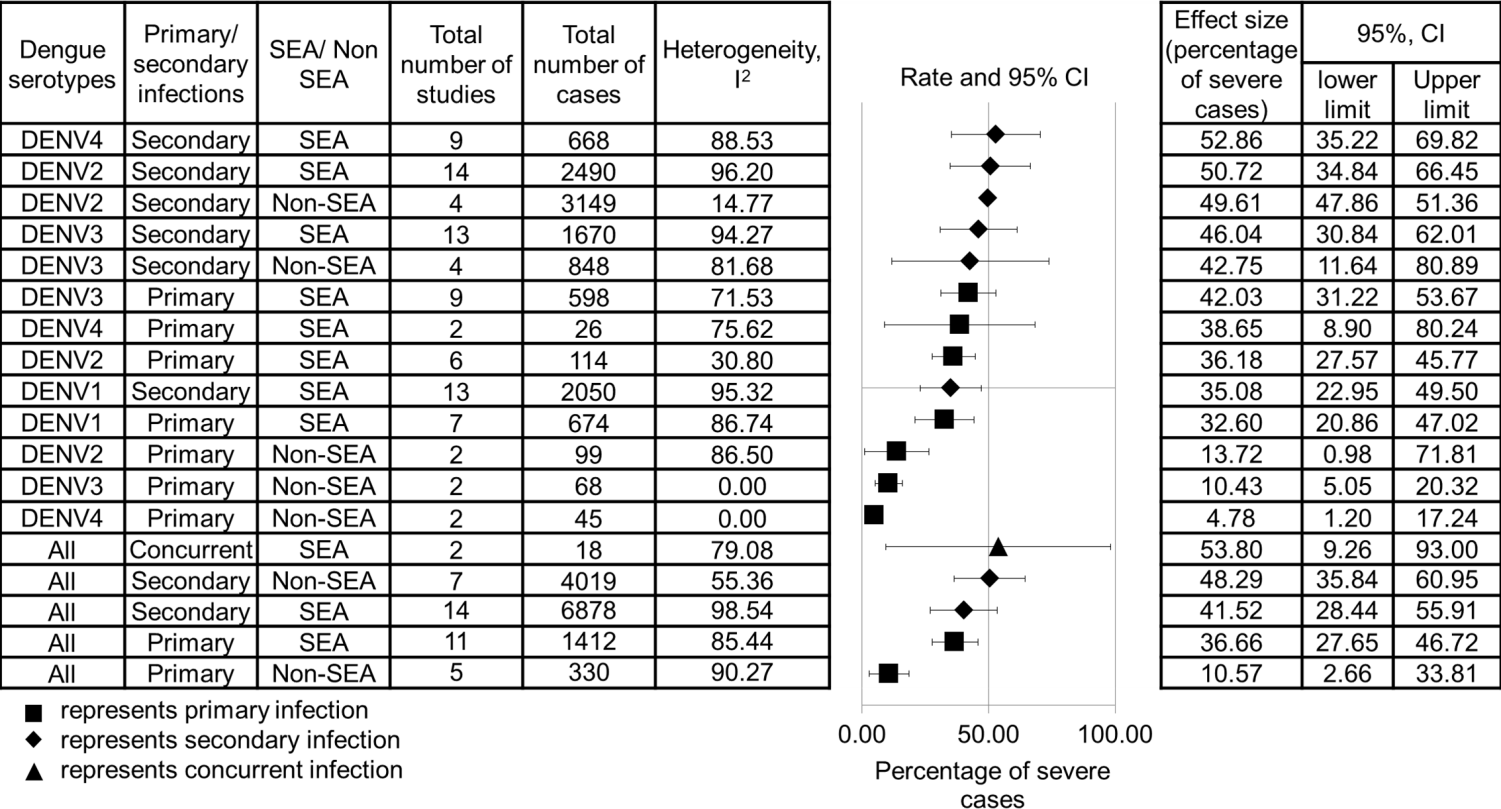
Pooled effect size of the relationship between dengue serotypes and percentage of severe cases. The data are arranged in a descending order based on the percentage of severe cases of dengue serotypes. The data are separated by primary and secondary infections, as well as SEA and non-SEA regions, regardless of the WHO classification.

The effect size of the percentage of severe dengue infections is shown in Fig 2. No meta-analysis was performed for primary DENV-1, secondary DENV-1 and secondary DENV-4 infections in non-SEA regions as it required more than one study. DENV-4 displayed the lowest total number of cases in both SEA and non-SEA regions (8.08% and 1.07%) compared to other serotypes. Hence, sensitivity analysis was carried out by omitting each individual study to observe its impact upon the quality and the consistency of the results. The results suggested that the study conducted by Nisalak et al [20] was reported to affect the heterogeneity of primary DENV-3 infection in the SEA region, while studies carried out by Guzman et al and Ocazionez et al [42,43] were reported to affect the heterogeneity of secondary DENV-3 infection in non-SEA regions. The heterogeneity did not remain significant after the studies were excluded. The remaining study groups contained no single study that significantly affected the effect size and quality.

Furthermore, the analysis (Fig 2) showed that secondary infection caused a greater percentage of severe infections than primary infection. When the data from all dengue serotypes were clustered into primary, secondary, and concurrent infections and were compared, concurrent infection was found to have higher percentage of severe cases than secondary infection, followed by primary infection. For primary infection, DENV-3 from the SEA region resulted in the highest percentage of severe infections, whereas DENV-2 and DENV-3 from non-SEA regions, as well as DENV-2, DENV-3 and DENV-4 from the SEA region, resulted in greater percentages of severe infections for secondary infection.

When the severity of dengue infections from the SEA region was compared with that of non-SEA regions (Peru, Cuba, France, Colombia, and Brazil), except secondary infections by DENV-2 and DENV-3; all primary and secondary dengue infections had lower percentage of severe cases in non-SEA regions than in the SEA region.

Moreover, DENV-2 and DENV-4 from the SEA region (95% CI, 14.21–14.54), as well as DENV-2 and DENV-3 from non-SEA regions (95% CI, 32.32–35.89), showed a greater difference in the percentage of severe dengue infections between primary and secondary infections compared to other serotypes.

**Fig 3:**
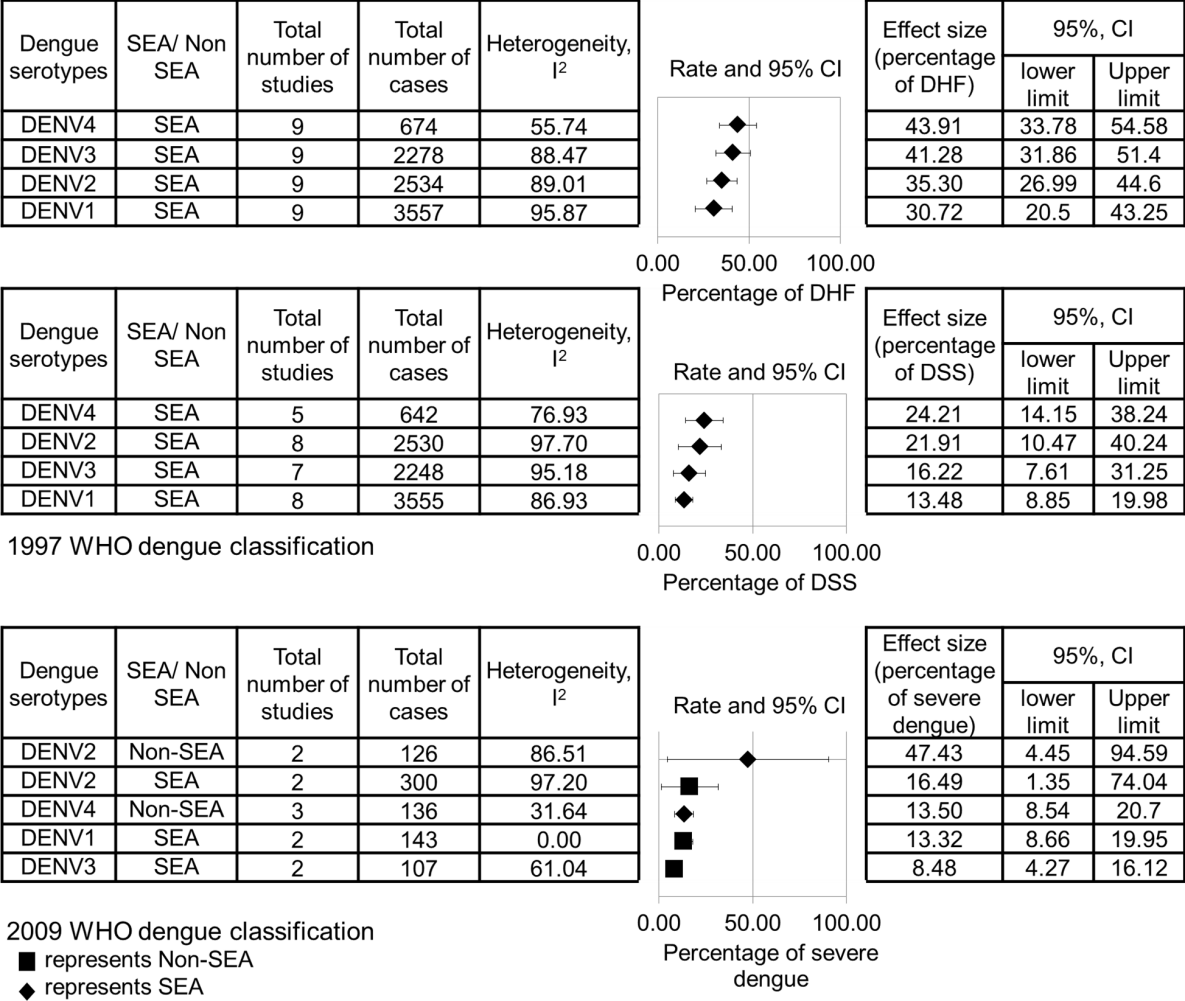
Pooled effect size of the relationship between dengue serotypes and percentage of DHF, DSS, as well as severe dengue infections. The data are arranged in a descending order based on the percentages of DHF, DSS and severe dengue infections. The data are separated by 1997 and 2009 WHO dengue classifications.

In addition, the effect size of the percentages of DHF, DSS and severe dengue infections is shown in Fig 3. According to 1997 WHO classification, DENV-3 and DENV-4 were found to result in higher percentage of DHF, whereas DENV-2 and DENV-4 had higher percentage of DSS, compared to other serotypes. According to 2009 WHO classification, DENV-2 from non-SEA regions were found to result in higher percentage of severe dengue infections than other serotypes.

### 3.3 The relationship between dengue serotypes and secondary infection

**Fig 4:**
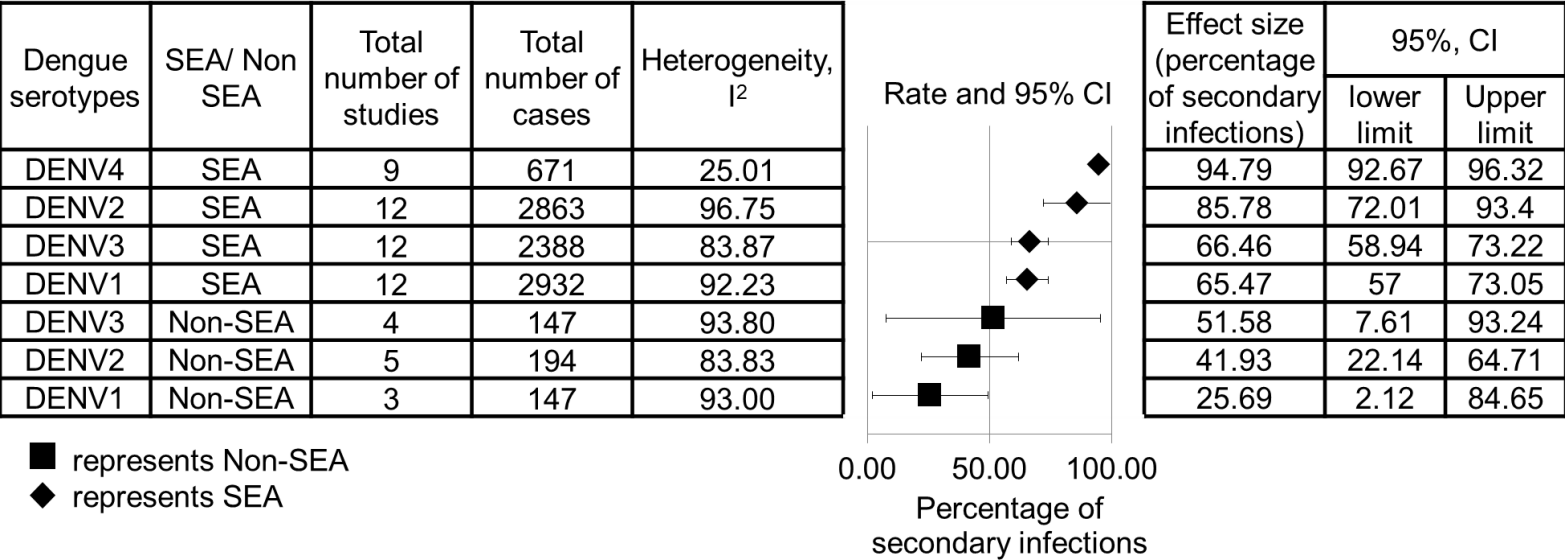
Pooled effect size of the relationship between dengue serotypes and secondary infection. The data are separated by SEA and non-SEA regions.

The effect size of the percentage of secondary infection is shown in Fig 4. DENV-2 and DENV-4 from the SEA region were found to result in higher percentages for secondary infection than other serotypes (95% CI, 72.01–96.32, 9–12 studies, n = 671–2863, I^2^ = 25.01–96.75%). Moreover, the percentage of secondary infection in the SEA region had been found to be higher than that of non-SEA regions. Moreover, results derived from the sensitivity analysis suggested that a study conducted by Dussart et al [41] was reported to affect the heterogeneity of DENV-2 infections in SEA regions and DENV-3 infections in non-SEA regions, while a study carried out by Libraty et al [24] was reported to affect the heterogeneity of DENV-3 infections in the SEA region. The heterogeneity did not remain significant after these studies were excluded. The remaining study groups contained no single study that significantly affected the effect size and quality.

### 3.4 Results of publication bias assessment

**Fig 5:**
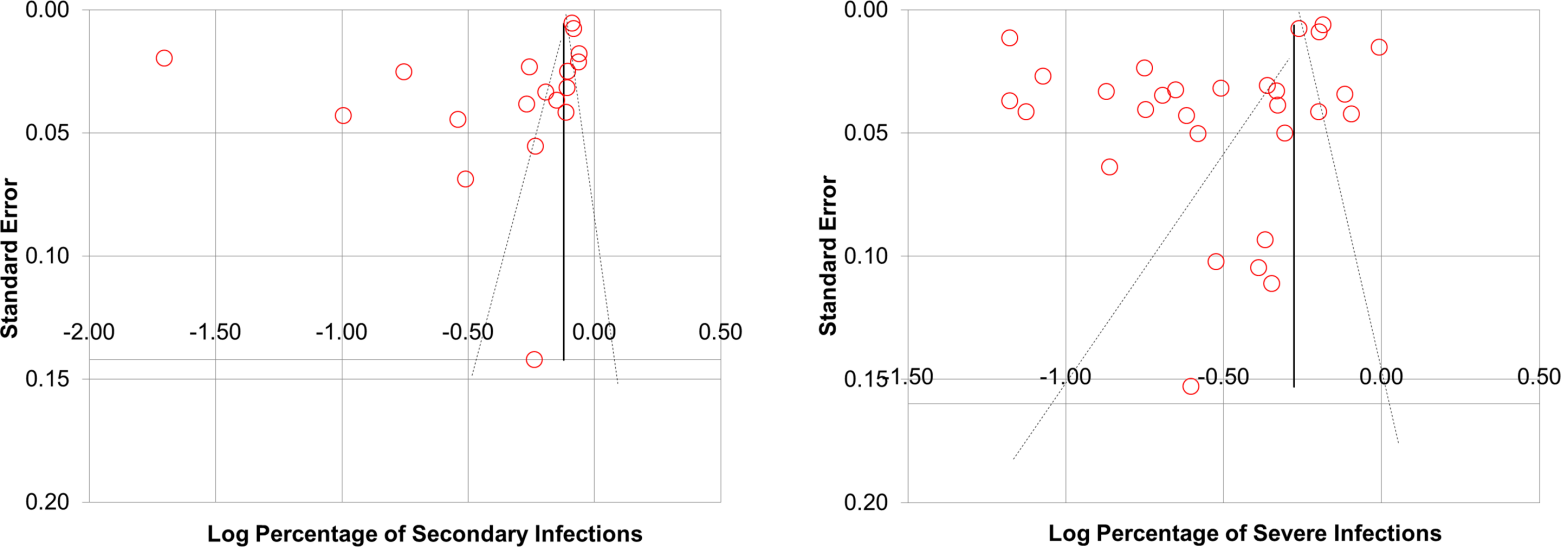
Funnel plot of the percentages of severe infections against standard error and funnel plot of the percentages of secondary infection against standard error. The percentages of severe infections are displayed on a logarithmic scale. The shapes of both the funnel plots revealed obvious asymmetry.

When analyzing all 29 studies that examined the association between dengue serotypes and severity, publication bias was detected by using Egger’s test (p = 0.000 (<0.05)), but not detected by using Begg’s test (p = 0.8219 (>0.05)). Besides, for all 18 studies that examined the association of secondary infection, publication bias was detected by using Egger’s test (p = 0.007 (<0.05)), but not detected by using Begg’s test (p = 0.211 (> 0.05)) (Fig 5).

## Discussion

Previous meta-analyses have addressed the epitope characteristics [39] and the host metabolism of dengue virus [40]:- the association between clinical signs [44] or temperature change [45] and the risk of dengue infection:- as well as current treatments for dengue infection such as the tetravalent vaccine [46], steroids [47] and supportive treatments such as fluid bolus [48] or vector control measures [49,50]. Nonetheless, this meta-analysis compared the percentage of severe cases of different serotypes of dengue virus in primary and secondary infections. Data from previous studies were collected and combined to achieve a sufficient sample size from both primary and secondary infections by each dengue serotype.

### 4.1 The association between dengue serotypes and severity

Secondary infection by each serotype showed greater percentage of severe cases than the respective primary infection (Fig 2). This strengthens the evidence that antibody-dependent enhancement occurs during secondary infection, which causes more severe infections.

Meanwhile, concurrent infection with dual serotypes is defined as a simultaneous infection with two serotypes of the dengue virus. The results showed that concurrent infections had greater percentage of severe cases than secondary infections and primary infections (Fig 2), although the difference was small (about 5%). Moreover, Guzman et al and Anderson et al [42,51] discovered that the longer the interval between the primary and secondary infections, the greater the risk of DHF. Hence, this finding suggests that concurrent infection with multiple serotypes, which represents zero time interval between primary and secondary infections, may present greater severity than both primary and secondary infections. However, as this study only managed to obtain two concurrent infections reported with the percentage of severe cases from SEA regions, the sample size needs to be increased in the future before any conclusion can be made.

According to the 1997 WHO classification, percentage of severe cases of two serotypes were found to be 8–10% higher than other serotypes, whereas according to the 2009 WHO classification, percentage of severe cases of one serotype (DENV-2 from non-SEA regions) was found to be 30% higher than other serotypes. This suggests that 2009 WHO dengue classification showed clearer difference in association of dengue serotypes to the percentage of severe cases. Besides, it was further reported that the 2009 WHO dengue classification is better at representing levels of disease severity [52]. This finding also suggests that the use of different dengue classifications affects the clinical prediction of severity caused by each serotype.

### 4.2 The association between dengue serotypes and secondary infections

The SEA region depicted a higher percentage of secondary dengue infection than non-SEA regions (Fig 4), particularly DENV-2 and DENV-4 infections. This finding may be due to the presence of different genotypes in these regions. In particular, the DENV-2 American and Asian/American genotypes have been commonly reported in non-SEA regions [53–55], while the DENV-2 Asian I, II, cosmopolitan and American Asian genotypes have been commonly reported in the SEA region [53,56–58]. The DENV-2 American genotype is less virulent during secondary infection [59], and may cause patients to not present clinically, therefore lowering the number of secondary cases recorded. Moreover, the DENV-2 Asian genotype was found to be more associated with secondary infection compared to the DENV-2 American/Asian genotype [56].

Nevertheless, studies performed in 2002–2008 in the European Network for Diagnosis of Imported Viral Diseases (ENIVD) showed that the presence of the dengue virus 2 Asian I, II and cosmopolitan genotypes, as well as all dengue virus 4 genotypes (I, II, III, IV and cosmopolitan) among travelers from SEA to Europe. Besides, spreading of the Asian genotypes has been reported in a few other studies in countries in South America (Colombia, Brazil, and Venezuela) [34,43].

In addition, a higher percentage of secondary infection may also be due to the higher prevalence of dengue outbreaks in the SEA region than non-SEA regions (South America and Europe), which has caused many patients to have a history of past infections. Although there was no significant difference between the number of dengue cases per person and the regions (95% CI SEA-0.1; non-SEA-0.3) from 2002–2015, based on the data obtained from previous studies ([60–70], data shown S1 Supporting Information sheet 4 Supporting Information sheet 4), many more dengue cases remained unreported. Moreover, the SEA region displayed a higher percentage of secondary infection, although the majority of studies included in SEA reflected belong to infants, who were less likely to have a history of past infections (Table 1). This suggests that dengue cases may be more prevalent in the SEA region, as the populations have been proven to experience dengue infection at a young age.

Meanwhile, DENV-4 portrayed the lowest percentage of the sample size among all four serotypes in both SEA and non-SEA regions (8.08% and 1.07%, according to Fig 2). Genotypes I and II of DENV-4 showed negative correlations with each other, suggesting an internal competition between the genotypes [71]. Moreover, another study reported that DENV-4 is transmitted through the *Aedes albopictus* mosquito, a different species from other serotypes (*Aedes aegypti*) that dwells in areas away from the urban areas with lower human population, and hence, causes a lower rate of transmission [72,73]. The reasons underlying the reduced circulation of DENV-4, in fact, require further investigation.

### 4.3 Limitations

Throughout the study, only a small portion of data from non-SEA regions (Cuba, Peru, France, Colombia and Brazil) had been obtained. Many studies were excluded because they did not separate the primary from the secondary cases of dengue infections caused by each dengue serotype. Some studies were excluded because there were no DHF or severe dengue cases recorded. Therefore, this data was removed to avoid problems in computation of the effect size as suggested by Sterne and Bradburn [74]. Hence, although the results (Fig 2) showed that secondary infection by DENV-2 and DENV-3 from non-SEA regions had higher percentage of severe cases than those in the SEA region, this does not imply that dengue infections in non-SEA regions are more severe. Torres and Castro [75] showed that only 3.4% of the sample size of 427,627 dengue patients in Latin America suffered from DHF, which is lower than the percentages shown in this study (10.57% and 48.29% in primary and secondary infections of all dengue serotypes, respectively). Use of different WHO classification was also found to affect the association between dengue serotypes and severe cases. More studies are needed in the future before any conclusions can be made. Moreover, publication bias was identified in this study, which suggests that more unpublished data are needed for more robust statistical results. On top of that, sensitivity analysis was performed, and studies that affected the consistency and the quality of the results were identified as they affected the robustness of the statistical results.

Besides, as studies included are retrospective studies, they present with inferior level of evidence compared to prospective studies. Details such as days of admission and age of patients in each dengue serotypes and in primary and secondary infections were only recorded in 6 and 12 studies, respectively, out of 31 studies included in this meta-analysis (data shown in S1 Supporting Information document sheet 6). Hence, no meta-regression was performed. Aging was found to be associated with co-morbidities that lead to impairments of kidney, liver and lung [76–78], that comprise the 2009 WHO classification definition of severe cases. Late admission of dengue cases and inadequate monitoring in hospital may also enhance severity of infections. These details are crucial as they may become confounding factors for the study of association between dengue serotypes, types of infections and percentage of severe dengue cases. Future studies that consider these confounding factors are needed.

### 4.4 Conclusion

As the severity of dengue infection had been found to be affected by the dengue serotypes involved and also the interval between the primary and secondary infections, these factors need to be considered when clinical prediction of the severity of dengue patients is being made. Moreover, as certain serotypes resulted in higher percentage of severe cases, such as secondary infection by SEA DENV-2, DENV-3, DENV-4, and non-SEA DENV-2, DENV-3 or primary infections by DENV-3 from SEA: these serotypes require proper clinical attention.

Moreover, since DENV-4 was found to cause the lowest percentage of the sample size; serotype-specific antiviral treatments may be more focused on the other serotypes, DENV-1, DENV-2 and DENV-3. Besides, as DENV-2 and DENV-4 had been more associated with secondary infection, patients with a history of past dengue infection should take extra precautions during outbreaks of DENV-2 and DENV-4 infections.

## Supporting Information

S1 Prisma Checklist Prisma 2009 checklist. (DOCX)

S1 Supporting Information Sheet 1 Relationship between dengue serotypes and percentage of severe cases Sheet 2 Relationship between dengue serotypes and percentage of DHF, DSS, as well as severe dengue infections Sheet 3 Relationship between dengue serotypes and secondary infection Sheet 4 Dengue cases in SEA and non-SEA regions Sheet 5 Publication bias Sheet 6 Age and days of admissions of patients
(XLSX)
